# In-Situ Construction of Anti-Aggregation Tellurium Nanorods/Reduced Graphene Oxide Composite to Enable Fast Sodium Storage

**DOI:** 10.3390/nano14010118

**Published:** 2024-01-03

**Authors:** Haiguo Hu, Jiarui Zhong, Bangquan Jian, Cheng Zheng, Yonghong Zeng, Cuiyun Kou, Quanlan Xiao, Yiyu Luo, Huide Wang, Zhinan Guo, Li Niu

**Affiliations:** 1 International Collaborative Laboratory of 2D Materials for Optoelectronics Science and Technology, Institute of Microscale Optoelectronics, Shenzhen University, Shenzhen 518060, China; huhaiguo916@szu.edu.cn (H.H.); 1800282021@email.szu.edu.cn (Y.Z.); wanghuide@szu.edu.cn (H.W.); 2 Material and Energy School, Guangdong University of Technology, Guangzhou 510006, China; 2112102119@mail2.gdut.edu.cn (J.Z.); 2112002042@mail2.gdut.edu.cn (B.J.); 3 Guangzhou Key Laboratory of Sensing Materials and Devices, Center for Advanced Analytical Science, School of Chemistry and Chemical Engineering, Guangzhou University, Guangzhou 510006, China; koucuiyun@e.gzhu.edu.cn (C.K.); 2112105089@e.gzhu.edu.cn (Y.L.); 4 School of Chemical Engineering and Technology, Sun Yat-sen University, Guangzhou 510006, China

**Keywords:** Te NR/rGO composite, solution method, anode material, sodium ion battery

## Abstract

Sodium-ion batteries (SIBs) as a replaceable energy storage technology have attracted extensive attention in recent years. The design and preparation of advanced anode materials with high capacity and excellent cycling performance for SIBs still face enormous challenges. Herein, a solution method is developed for in situ synthesis of anti-aggregation tellurium nanorods/reduced graphene oxide (Te NR/rGO) composite. The material working as the sodium-ion battery (SIB) anode achieves a high reversible capacity of 338 mAh g^−1^ at 5 A g^−1^ and exhibits up to 93.4% capacity retention after 500 cycles. This work demonstrates an effective preparation method of nano-Te-based composites for SIBs.

## 1. Introduction

Sodium-ion batteries (SIB) have become a highly competitive candidate battery for post-lithium-ion batteries because of the low cost of sodium resources, abundant reserves, and similar working principle to lithium-ion batteries [1,2,3]. Compared to Li^+^ (~0.76 Å), Na^+^ (~1.02 Å) has a larger radius, making it more difficult to diffuse into the electrode and typically resulting in slower kinetics and unstable ion insertion/de-insertion behavior [4,5,6]. Nowadays, the commercialization of SIBs is hindered by their low energy density and poor cycling stability [7,8,9,10]. It is crucial to develop efficient anode materials with high energy density, stable structure, and fast Na^+^ diffusion channels. Although many anode materials for SIBs have been studied, such as intercalation [11,12,13], organic [14,15,16], conversion [17,18,19], alloying [20,21,22], and conversion-alloying materials [23,24,25], there is still a need to modify the existing anode materials or develop advanced anode materials with high capacity and good cycling performance, which is of great significance to realizing their practical applications.

Tellurium (Te), as a member of the chalcogen family and has excellent electronic conductivity (2 × 10^2^ S cm^−1^), high density (6.2 g cm^−3^), high theoretical specific capacity (420 mAh g^−1^), and high theoretical volume capacity (2621 mAh cm^−3^), which makes it a promising anode material for SIBs [26,27,28,29]. Usually, nanoscale Te is loaded onto various carbon with the aim of enhancing its cycling stability by alleviating the shuttle effect of sodium polytelluride and suppressing the volume expansion of Te [30,31] while also inhibiting the uneven Na^+^ insertion/de-insertion behaviors in carbon materials [32,33,34,35]. In 2015, Zhang et al. synthesized amorphous nano Te@cablelike microporous carbon (nano-Te@C) composite with Te content of 70 wt% at 800 °C for 3 h [29]. The nano-Te@C composite was used as the anode material of a SIB, achieving a reversible capacity of 303 mAh g^−1^ at 0.8 A g^−1^ and a capacity retention of 90% after 1000 cycles at 0.06 A g^−1^. In 2018, Koketsu et al. prepared Te nanoparticle-infiltrated CMK-3-ordered mesoporous carbon (Te@CMK-3) composite containing 55wt% Te by calcining at 550 °C for 6 h [36]. This Te@CMK-3-based SIB anode obtained a specific capacity of 320 mAh g^−1^ at a current density of 0.8 A g^−1^. In 2019, Wang et al. prepared a Te nanoparticle-loaded carbon nanorod (Te/C_p_) composite with 67 wt% Te content at 480 °C for 12 h [37]. The Te/C_p_ composite, as the cathode material of the SIB, obtained a reversible specific capacity of 302 mAh g^−1^ at 0.8 A g^−1^. Then, Li et al. prepared a Te nanoparticle-loaded nitrogen-doped porous carbon sponge (NPCS/Te) composite with 50 wt% Te content at 480 °C for 6 h [38]. As the cathode material, this battery acquired a reversible specific capacity of 382 mAh g^−1^ at 0.2 A g^−1^. In 2022, Liu et al. prepared an amorphous Te-loaded CeO_2_ quantum dot/three-dimensional hierarchical porous carbon (Te/CeO_2_-QDs/HPC) composite with 62.5 wt% Te content at 480 °C for 20 h [34]. Using the Te/CeO_2_-QDs/HPC composite as the cathode material, the battery achieved a reversible specific capacity of 260 mAh g^−1^ at a high current density of 4 A g^−1^, and the specific capacity remained as high as 93.8% after 1000 cycles. However, these methods usually involve melting dispersion manufacturing processes with high energy consumption and are highly time-consuming. In addition, the reversible specific capacity and cycle stability of nano-Te-based SIBs need to be improved further.

In this work, an anti-aggregation Te nanorod/reduced graphene oxide (Te NR/rGO) composite is synthesized via an in situ solution method and its potential as an anode material for SIBs is explored. Firstly, the composite is synthesized via the solution method in a water bath of 50 °C for 3 h, avoiding the use of a high-temperature calcination operation, which saves energy consumption and reduces time. Secondly, the synthesized Te nanorods are evenly embedded in rGO and tightly combined with it to avoid Te agglomeration, which is beneficial for the composite to participate in the battery reaction. Thirdly, the introduction of the rGO material as a buffer layer can effectively accommodate the volume expansion of the Te material in the battery reaction process, which effectively prevents its crushing and aggregation in the cycle process. As a result, the synthesized anti-aggregation Te NR/rGO composite as the anode material of the SIB achieves a high reversible capacity of 338 mAh g^−1^ at a high current density of 5 A g^−1^ and shows a significant capacity retention rate of 93.4% after 500 cycles. This work provides an effective preparation method for nano-Te based composites for the practical application of SIBs.

## 2. Materials and Methods

### 2.1. Synthesis of the Te NR/rGO Composite

**Scheme 1:**
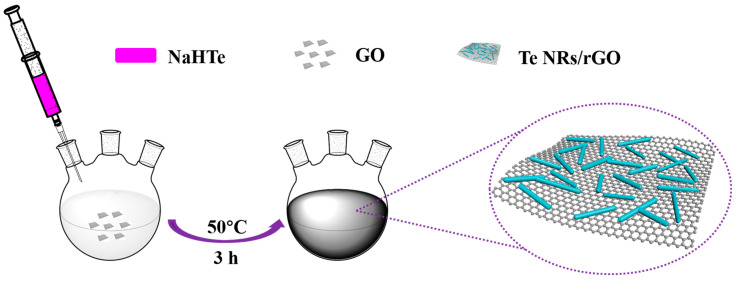
Illustration of the synthesis of the Te NR/rGO composite.

The Te NR/rGO composite was synthesized through an in situ solution method (depicted in Scheme 1). First, 310 mg of PVP and 0.4 mL of GO aqueous solution (2 g L^−1^) were completely dispersed in 40 mL deionized water in a three-necked reaction flask, the atmosphere of which was replaced with argon gas via a series of gas and degas processes. Then, 0.3 mL of freshly synthesized NaHTe solution were quickly injected with an injector into the solution under vigorous magnetic stirring. After 3 h reaction in a water bath, the resulting hydrogel was centrifuged at 10,000 rpm for 5 min, washed three times with deionized water, and finally freeze-dried for 24 h to produce Te NR/rGO powders. A series of composites with different amounts of PVP (310, 620, and 1240 mg) were synthesized and denoted as Te NR/rGO-310, Te NR/rGO-620, and Te NR/rGO-1240. For comparison, the composite without PVP under the same other conditions was synthesized and denoted as Te/rGO.

### 2.2. Structural Characterization

The morphology of the synthesized material was observed by a Thermo APREO S scanning electron microscope (SEM) (Thermo Scientific, Waltham, MA, USA) at an acceleration voltage of 5 kV and a HITACH HT7700 transmission electron microscope (TEM) (HITACHI, Tokyo, Japan) at an acceleration voltage of 80 kV. High-resolution TEM (HRTEM) images and energy-dispersive X-ray spectroscopy (EDX) elemental mapping results of the composites were obtained with a JEOL F200 field emission transmission electron microscope (JEOL, Tokyo, Japan) with an acceleration voltage of 200 kV. Raman spectroscopy was measured with a Horiba Confocal Raman system model XploRA Plus spectrometer equipped with a 532 laser with 100 mW, and the acquisition time for each measurement was 15 s. X-ray diffraction (XRD) was carried out using a Bruker D8 Advance X-ray diffractometer (λ = 0.154 nm, Bruker, Karlsruhe, Germany) with a copper target and an angular range of 5–80° at a scanning rate of 5°/min. The operating current and voltage were 40 mA and 40 kV, respectively. Thermogravimetric analysis (TGA) was used to test the content of Te on a TGA4000 (PerkinElmer, Waltham, MA, USA) in air from 30 to 900 °C at a heating rate of 10 °C min^−1^. X-ray photoelectron spectroscopy (XPS) was performed on a Thermo Scientific K-Alpha instrument (Thermo Scientific, Waltham, MA, USA) with an Al Kα X-ray source and corrected with C 1s at 284.6 eV as an internal standard. The absorption spectra with a range of 200–1100 nm were obtained by a UH4150 spectrophotometer (HITACHI, Tokyo, Japan).

### 2.3. Electrochemical Measurements

The electrochemical properties of the composites were measured by assembling them into a coin cell. The electrode was prepared by coating the mixture of 60% active material, 30% superconducting carbon black, and 10% polytetrafluoroethylene binder onto a porous Cu foam substrate. The coin cell (2032 type) was assembled under an argon glove box with H_2_O and O_2_ content below 0.1 ppm using the Te NR/rGO composites as the working electrode and Na foil as the counter electrode. The electrolyte used was 1 M sodium hexafluorophosphate (NaPF_6_) in diglyme (100 vol%). The galvanostatic discharge/charge test was conducted on a Land cycler (Wuhan Kingnuo Electronic Co., Wuhan, China). Cyclic voltammetry (CV) was carried out on a CHI760E (CH Instruments, Shanghai, China) electrochemical workstation with a scan rate of between 0.05 V and 3 V. Electrochemical impedance spectroscopy (EIS) was tested on the CHI760E equipment with amplitudes ranging from 100 kHz to 100 mHz and an AC voltage of 5 mV.

## 3. Results

The Te NR/rGO composites were synthesized via an in situ oxidation-reduction solution method (Scheme 1) in which NaHTe served as both the reducing agent and the Te source and GO served as the oxidant and the loaded substrate material. Firstly, the aqueous solution of NaHTe was prepared via a previously reported method (see ESI for details) [39]. Then, the NaHTe solution was injected into a GO aqueous solution containing surfactant PVP under an argon atmosphere at 50 °C. After 3 h, the color of the solution changed from light yellow to brownish black, and the final powder product was collected by freeze-drying after three washing processes (see the Materials and Methods section and Figure S1a for details).

**Figure 1:**
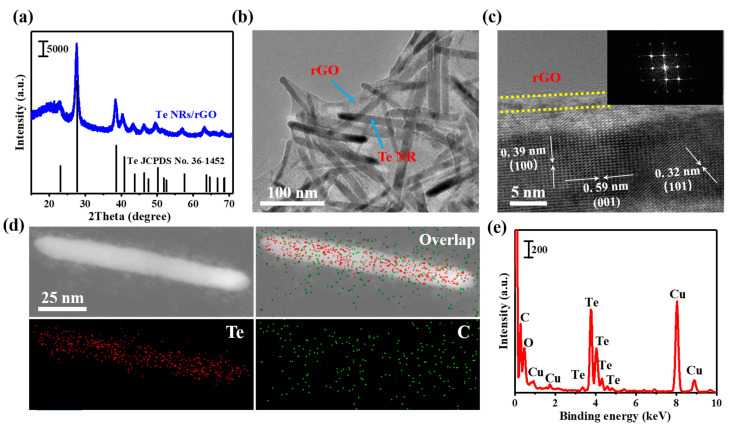
Structure and morphology characterization of Te NR/rGO-310. (**a**) XRD pattern, (**b**) TEM image, (**c**) HRTEM image (the inset is its corresponding FFT image), (**d**) elemental mapping images, (**e**) EDX spectrum.

XRD, SEM, and TEM were applied for characterization of the structure and morphology of Te NR/rGO-310. As shown in Figure 1a, the XRD curve of the Te NR/rGO composite was perfectly in accord with the characteristic peak of pure Te (JCPDS card No. 36-1465). The wide peak from 15 to 26° corresponded to carbon material and the sharp peaks contributed to Te, indicating that the Te materials in the composite had good crystallinity [40]. The SEM image (Figure S1b) clearly shows that the Te nanorods were successfully synthesized. From the TEM image exhibited in Figure 1b, it can be seen that the Te nanorods were tightly embedded in rGO and did not agglomerate. As shown in the HRTEM image of Te NR/rGO composite in Figure 1c, clear lattice spacing of 0.39, 0.59, and 0.32 nm contributed well to the (100), (001), and (101) faces of Te, respectively [41], which is strong evidence for the successful synthesis of the Te nanorods, consistent with the XRD results. Moreover, it can clearly be seen that the rGO of about 3 nm was tightly wrapped around the edge of the Te nanorods and the Te nanorods grew in the [001] direction. In addition, the sharp points in the corresponding fast Fourier transform (FFT) image confirm the high crystallinity of the Te nanorods (Figure 1c inset) [42]. The corresponding element mapping and EDX images of the Te NR/rGO composite in Figure 1d,e further confirm that the Te element was evenly distributed in the rGO, which is beneficial for the following electrode kinetics for sodium storage.

**Figure 2:**
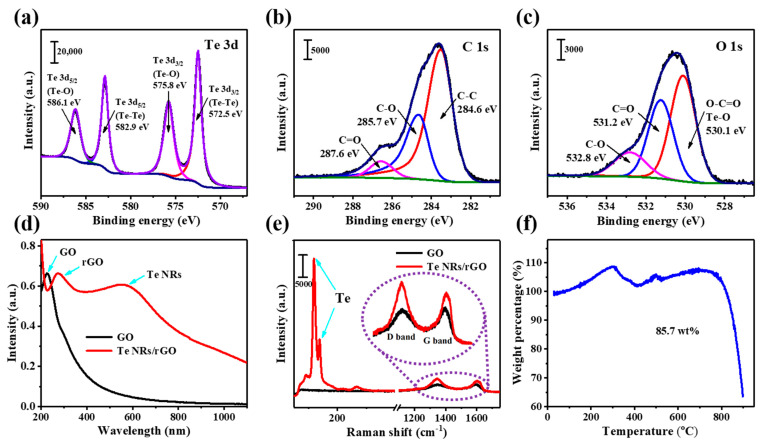
XPS spectra of (**a**) Te 3d, (**b**) C 1s, and (**c**) O 1s of Te NRs/rGO-310; (**d**) UV–vis–NIR absorption spectra of GO and Te NR/rGO-310; (**e**) Raman spectra of GO and Te NR/rGO-310; (**f**) TGA curve of Te NR/rGO-310.

The composition and valence of Te NR/rGO-310 were further examined by XPS characterization. The survey scan of the Te NR/rGO-310 revealed the main peaks of Te, C, O, and N, indicating the high purity of the Te NR/rGO composite (Figure S2). The XPS spectra of single components are depicted in Figure 2a–c. The two peaks at 572.5 eV (Te 3d5/2) and 582.9 eV (Te 3d3/2) were designated as Te-Te bonds of zero-valent Te in the composite (Figure 2a). The other two peaks, at 575.8 eV (Te 3d5/2) and 586.1 eV (Te 3d5/2), corresponded to the Te-O bond, which indicates that there was a small amount of oxide on the surface of Te NR/rGO composite [43]. The C1s spectrum can be divided into C-C (284.6 eV), C-O (285.7 eV), and C=O (287.6 eV) characteristic peaks (Figure 2b) [44]. Compared with the C1s spectrum of GO (Figure S3a), the peak intensity of the carbon–oxygen bond of the Te NR/rGO composite was significantly reduced, which confirms the successful reduction of GO. The O1s peaks in Figure 2c at 530.1 eV, 531.2 eV, and 532.8 eV were attributed to the O=C-OH and the Te-O, C=O, and C-OH groups, respectively [45]. The peak intensity of the carbon–oxygen bond (532.8 eV) of Te NR/rGO-310 was significantly lower than that of GO (Figure S3b), which is consistent with the result of the C1s peak change.

In addition, the optical changes of GO before and after the redox reaction were investigated by UV–vis–NIR absorption spectroscopy. As shown in Figure 2d, the absorption peak of GO, which was about 227 nm before the reaction (black line), red-shifted to 267 nm (red line) after the reaction, indicating that GO was reduced to rGO. The new wide peak at ~570 nm indicates the formation of Te nanorods [46]. Raman spectroscopy was used to inquire into the composition and structure of Te NR/rGO composite (Figure 2e). The characteristic peaks observed at 120.0 cm^−1^ and 138.7 cm^−1^ corresponded to the A_1_ and E_2_ vibration modes of low-dimensional Te nanostructures, respectively. Two peaks at 1348 and 1595 cm^−1^ were assigned to the D band and G band of the GO, respectively [47]. Compared with GO, the G band of Te NR/rGO-310 was blue-shifted to 1601 cm^−1^, and the peak intensity ratio of the D band and G band (*I*_D_/*I*_G_) increased from 0.96 to 1.22. This proves again that GO was successfully reduced during the formation of Te nanorods with this solution method [48]. Thermogravimetry was used to measure the content of Te in composites under an air atmosphere (Figure 2f). The weight content of Te in Te NR/rGO-310 was approximately 85.7 wt%, which is higher than that of other reported Te/C composites (≤70 wt%) [29,34,36,37,38], indicating that synthesized composites may have more active catalytic sites for electrochemical reactions.

TEM and Raman spectra were used to analyze the changes in Te NR/rGO-310 at different reaction times. The TEM images of the sample under 1 h, 2 h, 3 h, and 6 h reaction times are shown in Figure S4, indicating that the size of the Te nanorods gradually increased with the increase in reaction time and did not changes when the reaction time exceeded 3 h. The *I*_D_/*I*_G_ value in the Raman spectra of the composites exhibited the same tendency as the TEM results (Figure S5). GO gradually decreased and Te nanorods gradually formed and grew with the increase in time. When the reaction time exceeded 3 h, the raw material was completely exhausted, at which point the reaction stopped and the Te nanorods would not grow any more.

**Figure 3:**
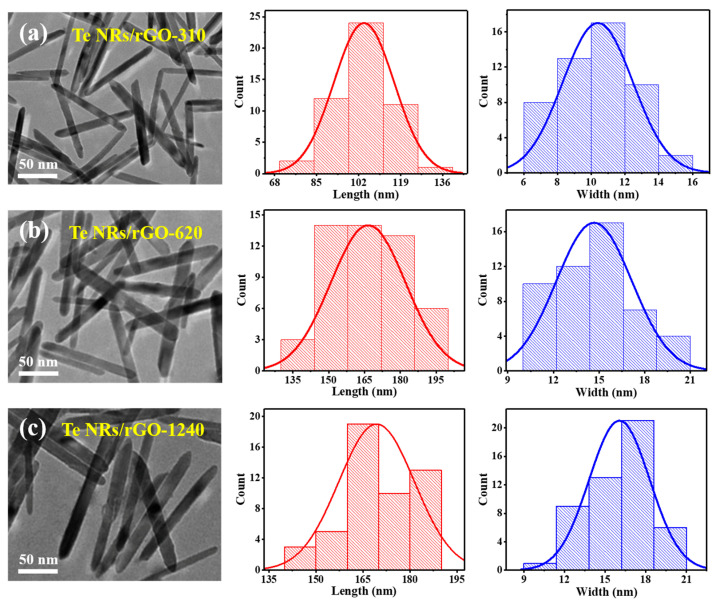
TEM images and corresponding length and width histograms of Te NR/rGO-310, Te NR/rGO-620, and Te NR/rGO-1240.

In order to explore the effect of surfactant PVP on the Te NR/rGO composites, comparative experiments were carried out with different amounts of PVP (620 mg and 1240 mg). The characterizations of XRD and XPS revealed that Te nanorods with good crystalline structure could be obtained in the Te NR/rGO composites under any amount of PVP (Figures S6–S8). The difference is that the morphology of the composites changed with the PVP content (Figure 3 and Table S1). The size of the Te nanorods in the Te NR/rGO composites increased with the PVP content. However, when the amount of PVP exceeded 620 mg, the size of the Te nanorods no longer increased. Statistical analysis of 50 Te nanorods showed that the length and diameter of the Te-NR/rGO-310 were 99.7 ± 17.8 nm and 14.3 ± 2.4 nm, respectively (Figure 3a). When the PVP content increased to 620 mg, the length of the Te nanorods increased to 166.7 ± 19.5 nm and the diameter increased to 16.6 ± 3.3 nm (Figure 3b). When the amount of PVP continued to increase to 1240 mg, the size of the Te nanorods saw little change compared with 620 mg PVP, with a length and diameter of 169.5 ± 21.4 nm and 17.1 ± 3.1 nm, respectively (Figure 3c). This indicates that the size of Te nanorods in composites can be controlled by adjusting the amount of PVP. In addition, it was found that Te nanorods could not be obtained without PVP under the same other conditions, but shapeless Te in micrometer size was obtained (Figure S9), indicating that the surfactant PVP was very important for the formation of Te in a nanorod structure during the reaction. The thermogravimetric test found that the weight content of Te nanorods in the Te NR/rGO composites decreased with the increase in PVP, which was 85.7 wt%, 80.8 wt%, and 80.0 wt% for samples with Te NR/rGO-310, Te NR/rGO-620, and Te NR/rGO-1240, respectively (Figure 2f and Figure S10). This might be because the excess PVP reduced the affinity between the Te NR and rGO, resulting in some dissociative Te NR, which would have been washed out during the washing process.

**Figure 4:**
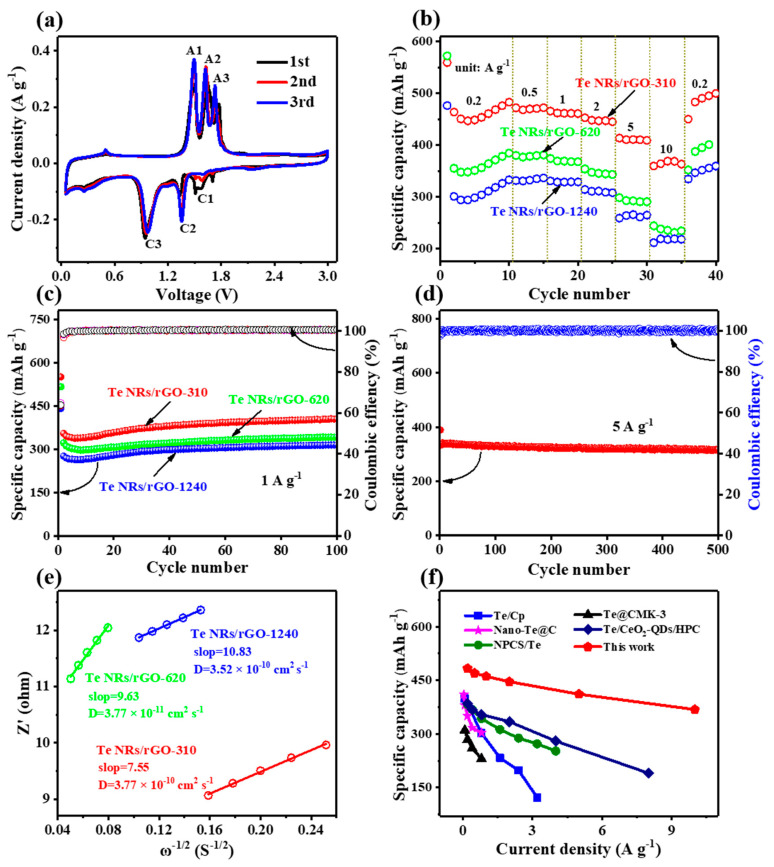
Electrochemical characterization of Te NR/rGO composite-based SIBs with a 1 M NaPF_6_ electrolyte in diglyme solvent. (**a**) CV curves of Te NR/rGO-310 at a scan rate of 0.2 mV s^−1^; (**b**) rate capability of Te NR/rGO-310 from 0.2 to 10 A g^−1^; (**c**) cycle performance of Te NR/rGO-310, Te NR/rGO-620, and Te NR/rGO-1240 at 1 A g^−1^; (**d**) long cycle stability of Te NR/rGO-310 at 5 A g^−1^; (**e**) linear fitting in the low-frequency region corresponding to the Nyquist plots of Te NR/rGO-310, Te NR/rGO-620, and Te NR/rGO-1240; (**f**) gravimetric specific capacity of the Te NR/rGO composite-based electrode compared with some representative electrodes for SIB, e.g., Te/Cp [37], Nano-Te@C [29], NPCS/Te [38], Te@CMK-3 [36], and Te/CeO_2_-QDs/HPC [34].

The sodium storage performance of the Te NR/rGO composite was evaluated in the half cells using Na metal as the counter electrode. Firstly, the CV analysis was performed at a scan rate of 0.2 mV s^−1^ to understand the redox behavior (Figure 4a). The CV curves showed a three-step process of redox reaction with three anodic peaks and three cathodic peaks. The C1, C2, and C3 peaks were related to the reduction in Te to Na_2_Te_6_, the continuous reduction of Na_2_Te_6_ to Na_2_Te_2_, and the final obtained Na_2_Te products, respectively [38], whereas the A1, A2, and A3 peaks corresponded to the reverse oxidation reaction from Na_2_Te to Te. Three nearly overlapping CV curves suggest that the Te NR/rGO-310 had good electrochemical reversibility. The rate performance of the Te NR/rGO composite are shown in Figure 4b and Figure S11. For Te NR/rGO-310, the specific capacities of 483, 470, 461, 446, 411, and 368 mAh g^−1^ were obtained at increasing current densities of 0.2, 0.5, 1, 2, 5, and 10 A g^−1^, respectively. Even under the ultra-high current density of 10 A g^−1^, the Te NR/rGO composite maintained a high specific capacity up to 368 mAh g^−1^, demonstrating excellent high-rate performance. When returning to the lowest current density of 0.2 A g^−1^, it completely reached the initial value and delivered about 489 mAh g^−1^, indicating that the Te NR/rGO electrode was stable under different current densities. It was also found that when the PVP content increased, the specific capacity of the Te NR/rGO composite did not continue to increase but rather declined (Figure 4b). After adding 620 mg PVP, the specific capacities of 385, 378, 368, 345, 292, 236, and 388 mAh g^−1^ were obtained at 0.2, 0.5, 1, 2, 5, 10, and 0.2 A g^−1^, respectively. When the amount of PVP reached 1240 mg, the specific capacities further declined to 333, 332, 329, 310, 265, 218, and 352 mAh g^−1^ under the same test conditions, respectively.

Figure 4c exhibits the cycling performance of the Te NR/rGO composites with different PVP content, all of which maintained good stability and kept a capacity retention of almost 100% over 100 cycles. The increase in specific capacity during the cycle may have been caused by the activation of electrode materials during the charge and discharge process. In addition, the Te-NR/rGO composite with 310 mg PVP offered a specific capacity of 391 mAh g^−1^ at 1 A g^−1^, which is higher than the other composites with 620 mg (330 mAh g^−1^) and 1240 mg (305 mAh g^−1^). It is worth noting that the Te-NR/rGO composite with 310 mg PVP delivered a high specific capacity of 338 mAh g^−1^ at a high current density of 5 A g^−1^ and maintained a capacity retention of 93.4% after 500 cycles (Figure 4d), showing a high rate-specific capacity and long-term stability. In contrast, the Te/rGO composite achieved only a low specific capacity of 251, 252, 251, 239, 211, 182, and 263 mAh g^−1^ at 0.2, 0.5, 1, 2, 5, 10, and 0.2 A g^−1^ current density, respectively (Figure S12a). This electrode only retained 74% of its specific capacity after 20 cycles at a current density of 5 A g^−1^, showing relatively poor stability (Figure S12b). Therefore, the synergistic effect between small-sized Te nanorods with even distribution and rGO resulted in the high specific capacity and long-term cycle stability of the Te NR/rGO anodes. EIS was used to further study the electrochemical characteristics of Te NR/rGO composites. It was found that the Te NR/rGO composites had very similar resistance values under different PVP amounts (Figure S13). The Na^+^ diffusion coefficients (*D*) of the composites were further calculated using the data from the Nyquist plots, as shown in Figure 4e [49]. It is clear that Te NR/rGO-310 had a significantly lower slope (7.55) and D value (3.77 × 10^−10^ cm^2^ s^−1^) than the other two composites. This is attributed to the tight combination between the smaller Te nanorods and rGO, which significantly enhanced electrochemical dynamics and excellent sodium storage performance. Figure 4f and Table S2 compare the performance of recently published Te-based electrode materials for SIBs. It can be seen that the Te NR/rGO composite of this work has obvious advantages, especially in the aspect of high rate performance. This Te NR/rGO-310 delivered 483 mAh g^−1^ at 0.2 A g^−1^, which is far higher than other reports [29,34,36,37,38]. In addition, at a current density of up to 5 A g^−1^, the specific capacity was still up to 338 mAh g^−1^, indicating that these Te NR/rGO composites had high rate performance.

In addition, the kinetic processes and capacitance contributions were analyzed based on the CV measurements and corresponding calculations at different scanning rates from 0.2 to 5.0 mV s^−1^ (Figure S14a). For the Te NR/rGO-310 composite, the b values of the three anode and two cathode peaks were calculated as 0.75, 0.87, 0.91, 0.81, and 0.81, respectively (Figure S14b), indicating that the storage of Na^+^ in the electrochemical process was mainly dependent on surface control [50]. In addition, the Te NR/rGO-310 composite showed a high capacitive contribution of 84%, 82%, 83%, 87%, and 98% at scan rates of 0.2, 0.5, 1.0, 2.0, and 5.0 mV s^−1^, respectively (Figure S14c,d). The surface capacitance behavior enhanced the electrochemical kinetics [51,52], which explains the excellent rate performance of the Te-NR/rGO composites.

## 4. Conclusions

In conclusion, this work used an in situ solution method at 50 °C for 3 h to synthesize anti-aggregation Te NR/rGO composites. It was found that PVP as a surfactant could promote the formation of Te nanorods along the [001] direction during the growth of the Te materials and make them evenly embedded in rGO. In addition, the synthesized Te NR/rGO-310 as the anode material of SIB showed superior specific capacity and cycle stability. The Te NR/rGO composite achieved a high reversible specific capacity of 338 mAh g^−1^ at a high current density of 5 A g^−1^ and exhibited a high specific capacity retention rate of 93.4% after 500 cycles. This work provides an effective preparation method of nano-Te-based composites for the practical application of SIBs.

## Data Availability

The data that support the findings of this study are available from the corresponding author (Z.G.) upon reasonable request.

## Supplementary Materials

The following supporting information can be downloaded at: https://www.mdpi.com/article/10.3390/nano14010118/s1, Figure S1: (a) Photographs of the GO and Te NR/rGO-310 aqueous solution; (b) SEM image of Te NR/rGO-310; Figure S2: XPS full spectrum of Te NR/rGO-310; Figure S3: XPS full spectrum, (a) C 1s and (b) O 1s of GO; Figure S4: TEM images of Te NR/rGO-310 at (a) 1 h, (b) 2 h, (c) 3 h, and (d) 6 h; Figure S5: Raman spectra of Te NR/rGO-310 at (a) 1 h, (b) 2 h, (c) 3 h, and (d) 6 h; Figure S6: XRD patterns of Te NR/rGO-310, Te NR/rGO-620, and Te NR/rGO-1240; Figure S7: XPS spectra of (a) full spectrum, (b) Te 3d, (c) C 1s, and (d) O 1s of Te NR/rGO-620; Figure S8: XPS spectra of (a) full spectrum, (b) Te 3d, (c) C 1s, and (d) O 1s of Te NR/rGO-1240; Figure S9: TEM image of Te/rGO composite; Figure S10: TGA spectra of Te NR/rGO-310, Te NR/rGO-620, and Te NR/rGO-1240; Figure S11: Charge–discharge profiles of Te NR/rGO-310 at different current densities from 0.2 to 10 A g^−1^; Figure S12: Electrochemical characterization of Te/rGO composite-based Na metal cells with a 1 M NaPF_6_ electrolyte in diglyme solvent. (a) Rate capability from 0.2 to 10 A g^−1^ and (b) long cycling performance at 5 A g^−1^; Figure S13: Nyquist plots of Te NR/rGO-310, Te NR/rGO-620, and Te NR/rGO-1240 composite-based SIB half-cell; Figure S14: Kinetic analysis of Te NR/rGO in SIB half-cell: (a) CV curves at scan rates from 0.2 to 5.0 mV s^−1^; (b) relationship between log *i* and log *ν* plots; (c) capacitive contribution at 0.5 mV s^−1^; (d) capacitance contribution at various scan rates; Table S1: Summary of nanorod length, width, and aspect ratio of Te NR/rGO-310, Te NR/rGO-620, and Te NR/rGO-1240 composites; Table S2: Summary of the representative nano-Te-based electrode materials for SIBs. References [29,34,36,37,38,39] are cited in the supplementary materials.
