# Supplementary material for: In-Situ Construction of Anti-Aggregation Tellurium Nanorods/Reduced Graphene Oxide Composite to Enable Fast Sodium Storage
Source: Nanomaterials (Basel). 2024 Jan 3;14(1):118. doi: 10.3390/nano14010118 (PMC10780675; doi:10.3390/nano14010118)
Supplement: Supplementary file 1 [file nanomaterials-14-00118-s001.zip › nanomaterials-2788098-supplementary.pdf]

In-Situ Construction of Anti-Aggregation Tellurium Nanorods/Reduced Graphene Oxide Composite to Enable Fast Sodium Storage

Haiguo Hu [1], Jiarui Zhong [3], Bangquan Jian [3], Cheng Zheng [3,*], Yonghong Zeng [1], Cuiyun Kou [2], Quanlan Xiao [1,*], Yiyu Luo [2], Huide Wang [1], Zhinan Guo [2,*] and Li Niu [4,*]

[1] International Collaborative Laboratory of 2D Materials for Optoelectronics Science and Technology,
Institute of Microscale Optoelectronics, Shenzhen University, Shenzhen 518060, China; huhaiguo916@szu.edu.cn (H.H.); 1800282021@email.szu.edu.cn (Y.Z.); wanghuide@szu.edu.cn (H.W.)
[2] Guangzhou Key Laboratory of Sensing Materials and Devices, Center for Advanced Analytical Science, School of Chemistry and Chemical Engineering, Guangzhou University, Guangzhou 510006, China; koucuiyun@e.gzhu.edu.cn (C.K.); 2112105089@e.gzhu.edu.cn (Y.L.)
[3] Material and Energy School, Guangdong University of Technology, Guangzhou 510006, China; 2112102119@mail2.gdut.edu.cn (J.Z.); 2112002042@mail2.gdut.edu.cn (B.J.)
[4] School of Chemical Engineering and Technology, Sun Yat-sen University, Guangzhou 510006, China
* Correspondence: guozhinan@gzhu.edu.cn (Z.G.); zhengcheng@gdut.edu.cn (C.Z.);
xiaoql@szu.edu.cn (Q.X.); niuli@mail.sysu.edu.cn (L.N.)

Supporting Information

Experiment

Materials

Bulk tellurium powder (99.99%, 200 mesh) was obtained from Macklin. Polyvinylpyrrolidone (PVP, MW ca. 58000) and sodium borohydride ($NaBH_4$, 98%) were purchased from Aladdin. Unless otherwise noted, all solvents used were of analytical reagent grade without further purification. Deionized water was produced from a Milli-Q water purification system.

Preparation of Sodium Hydrogen Telluride (NaHTe)

NaHTe was synthesized through a simple redox reaction [39]. Briefly, 0.34 g of $NaBH_4$ was dissolved in 6 mL of deionized water and then 0.51 g of Te powder was quickly added. Thereafter, the reaction vessel was sealed with a rubber stopper. Besides, a small pinhole was provided on the rubber stopper to ensure that H_2 generated by the reaction is discharged in time. During the reaction, the system was stirred and cooled in an ice-water bath to allow the reaction to proceed uniformly and slowly. After about 8 h, the black Te powder disappeared and a white sodium tetraborate precipitate was produced. The upper clear supernatant after stopping stirring for a while was the prepared NaHTe solution.

Results and Discussion

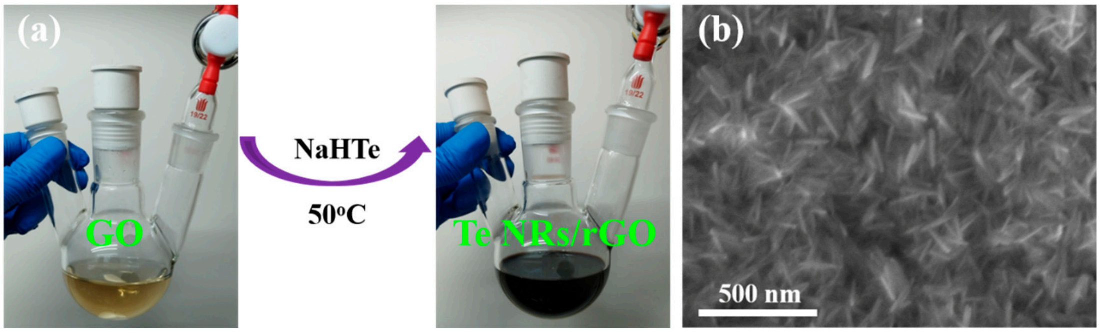

Figure S1. (a) Photographs of the GO and Te NRs/rGO-310 aqueous solution; (b) SEM image of Te NRs/rGO-310.

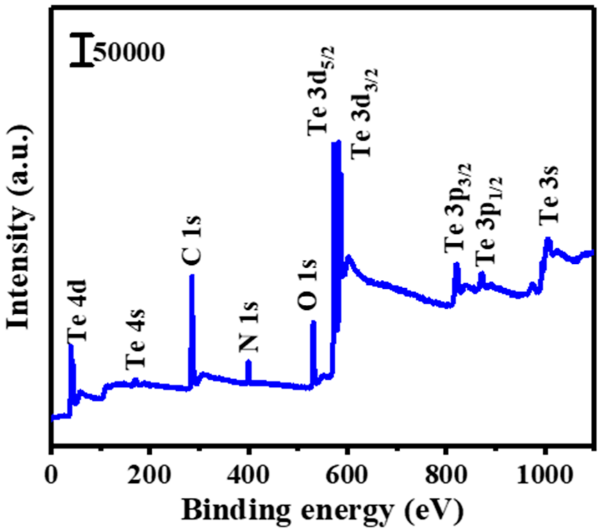

Figure S2. XPS full spectrum of Te NRs/rGO-310.

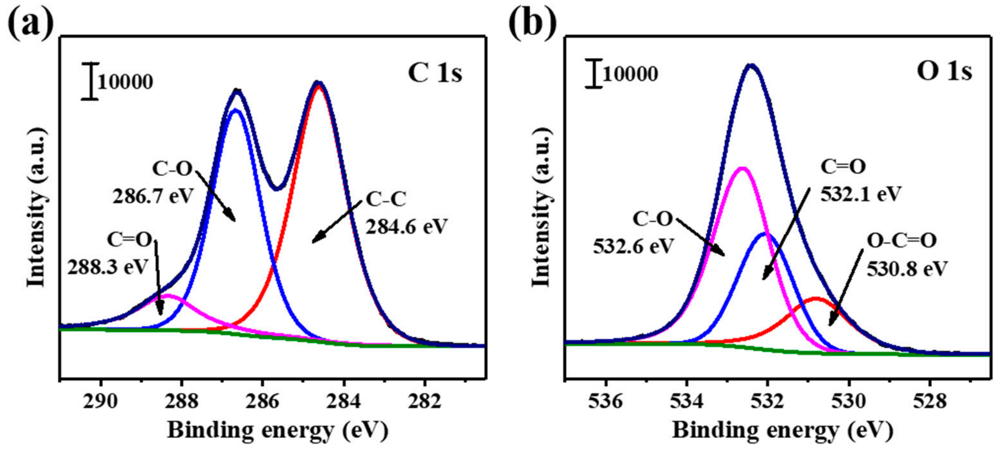

Figure S3. XPS full spectrum, (a) C 1s and (b) O 1s of GO.

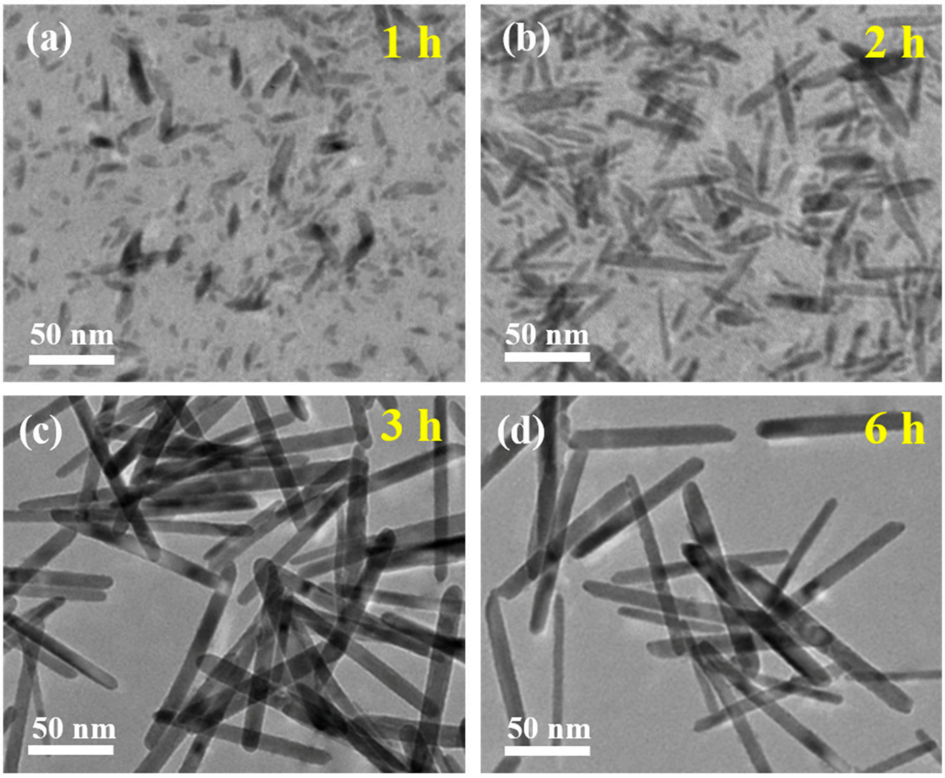

Figure S4. TEM images of Te NRs/rGO-310 at (a) 1 h, (b) 2 h, (c) 3 h and (d) 6 h.

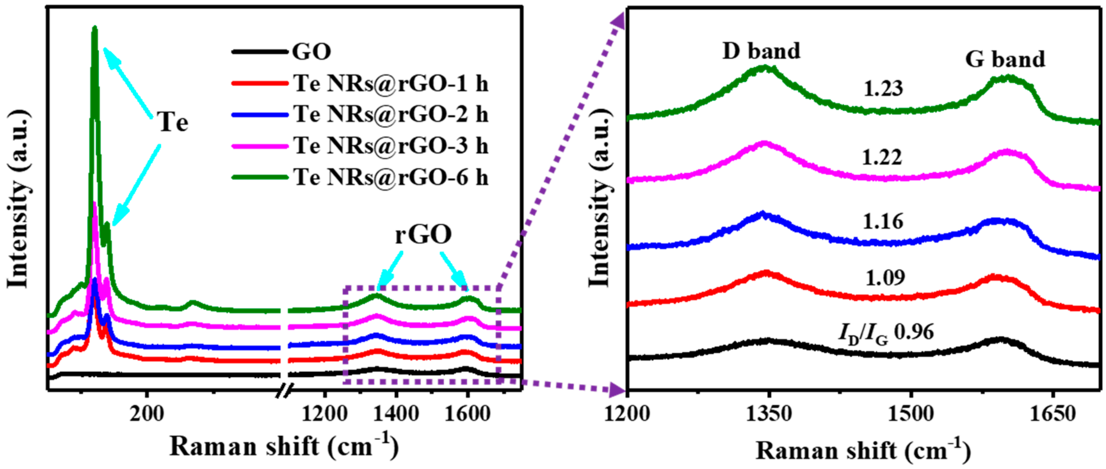

Figure S5. Raman spectra of Te NRs/rGO-310 at (a) 1 h, (b) 2 h, (c) 3 h and (d) 6 h.

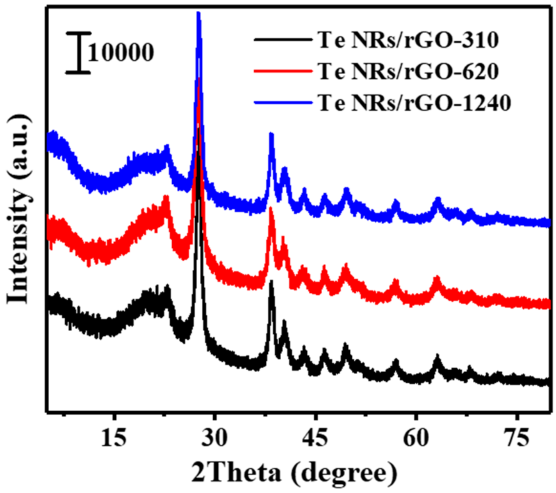

Figure S6. XRD patterns of Te NRs/rGO-310, Te NRs/rGO-620 and Te NRs/rGO-1240.

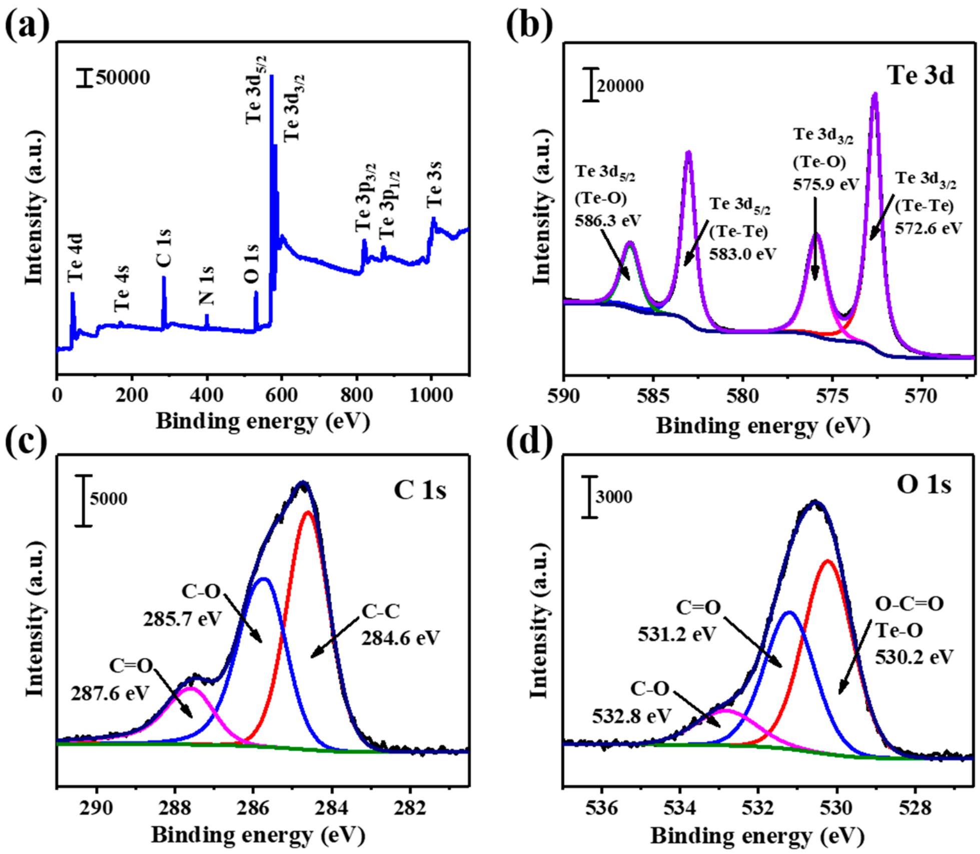

Figure S7. XPS spectra of (a) full spectrum, (b) Te 3d, (c) C 1s, and (d) O 1s of Te NRs/rGO-620.

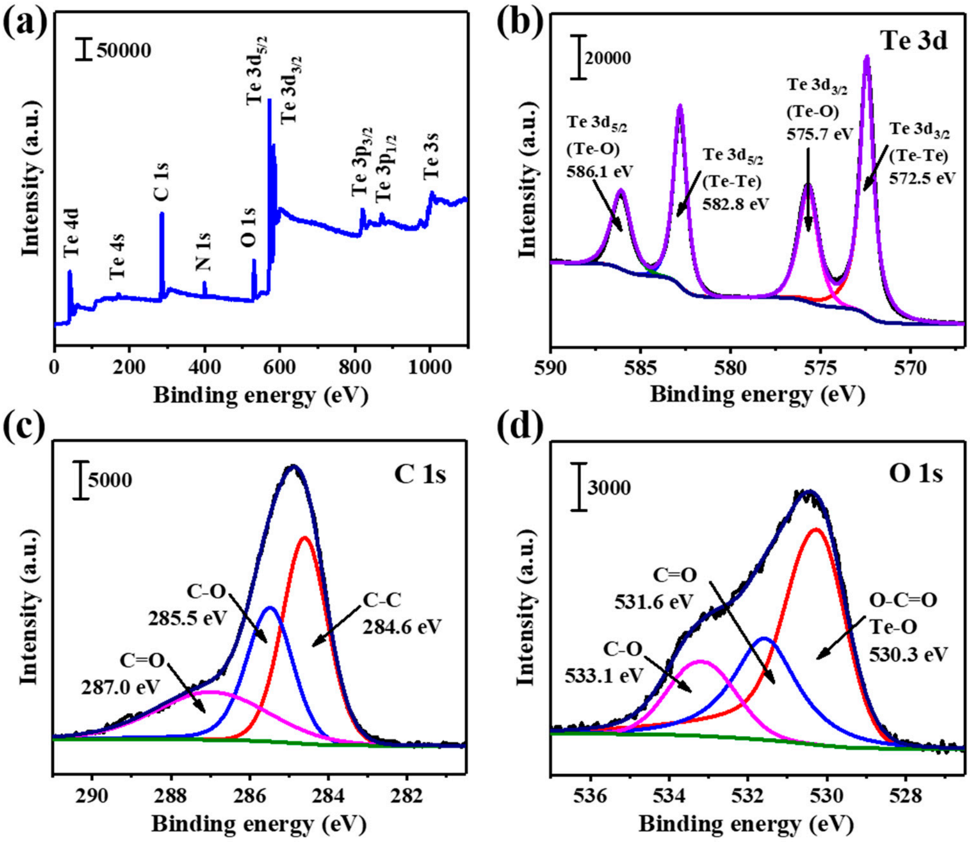

Figure S8. XPS spectra of (a) full spectrum, (b) Te 3d, (c) C 1s, and (d) O 1s of Te NRs/rGO-1240.

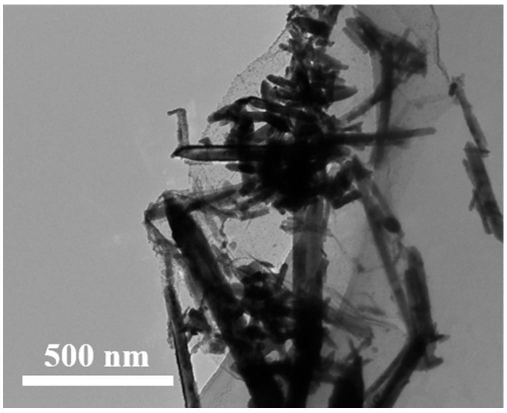

Figure S9. TEM image of Te/rGO composite.

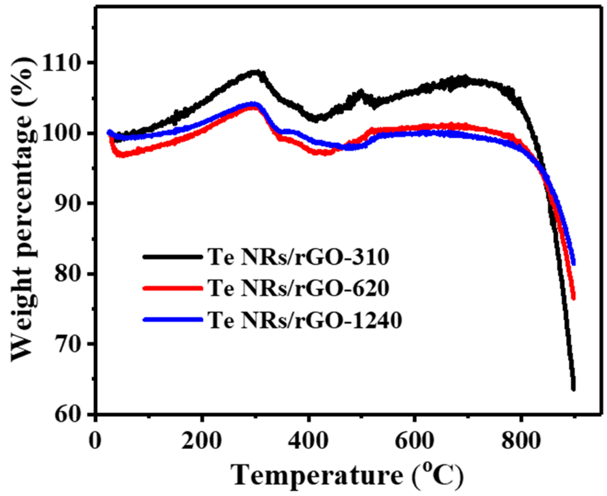

Figure S10. TGA spectra of Te NRs/rGO-310, Te NRs/rGO-620 and Te NRs/rGO-1240.

All the Te NRs/rGO composites were analyzed by TGA in the air to burn the rGO and leave Te as TeO_2 in the ceramic pan. The weight change from 25 to 900°C is a combination of weight loss from the rGO oxidation to carbon dioxide, $C + O_2 = CO_2$, and weight increase due to the oxidation of tellurium, $Te + O_2 = TeO_2$. And the Te content was determined by using the following equation:

$$Te\ (wt\%) = \frac{molecular\ weight\ of\ Te}{molecular\ weight\ of\ TeO_2} \times \frac{final\ weight\ of\ TeO_2}{initial\ weight\ of\ Te\ NRs/rGO} \times 100$$

Using the above equation, the weight content of Te nanorods in Te NRs/rGO-310, Te NRs/rGO-620 and Te NRs/srGO-1240 is 85.7 wt%, 80.8 wt%, and 80.0 wt%, respectively.

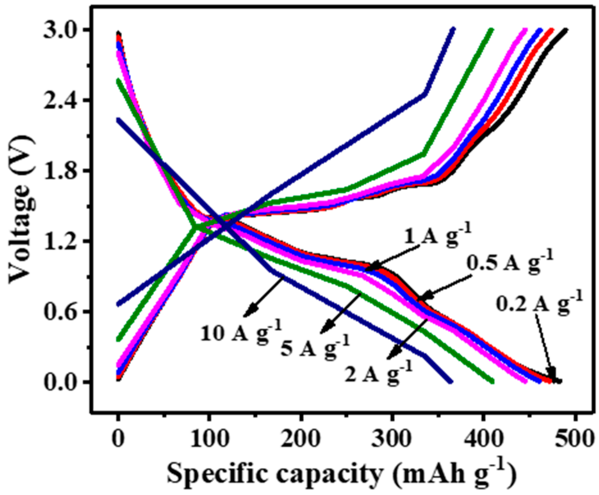

Figure S11. Charge-discharge profiles of Te NRs/rGO-310 at different current densities from 0.2 to 10 A g^{-1}.

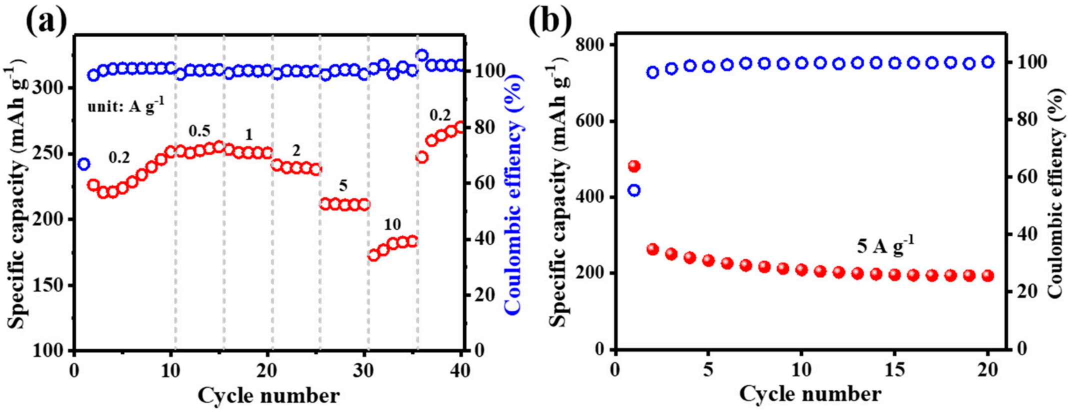

Figure S12. Electrochemical characterization of Te/rGO composite based Na metal cells with a 1 M $NaPF_6$ electrolyte in diglyme solvent. (a) Rate capability from 0.2 to 10 A g^{-1} and (b) long cycling performance at 5 A g^{-1} .

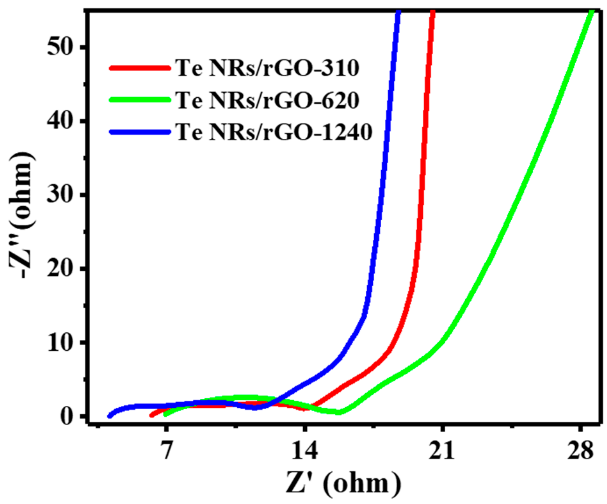

Figure S13. Nyquist plots of Te NRs/rGO-310, Te NRs/rGO-620 and Te NRs/rGO-1240 composites-based SIBs half-cell.

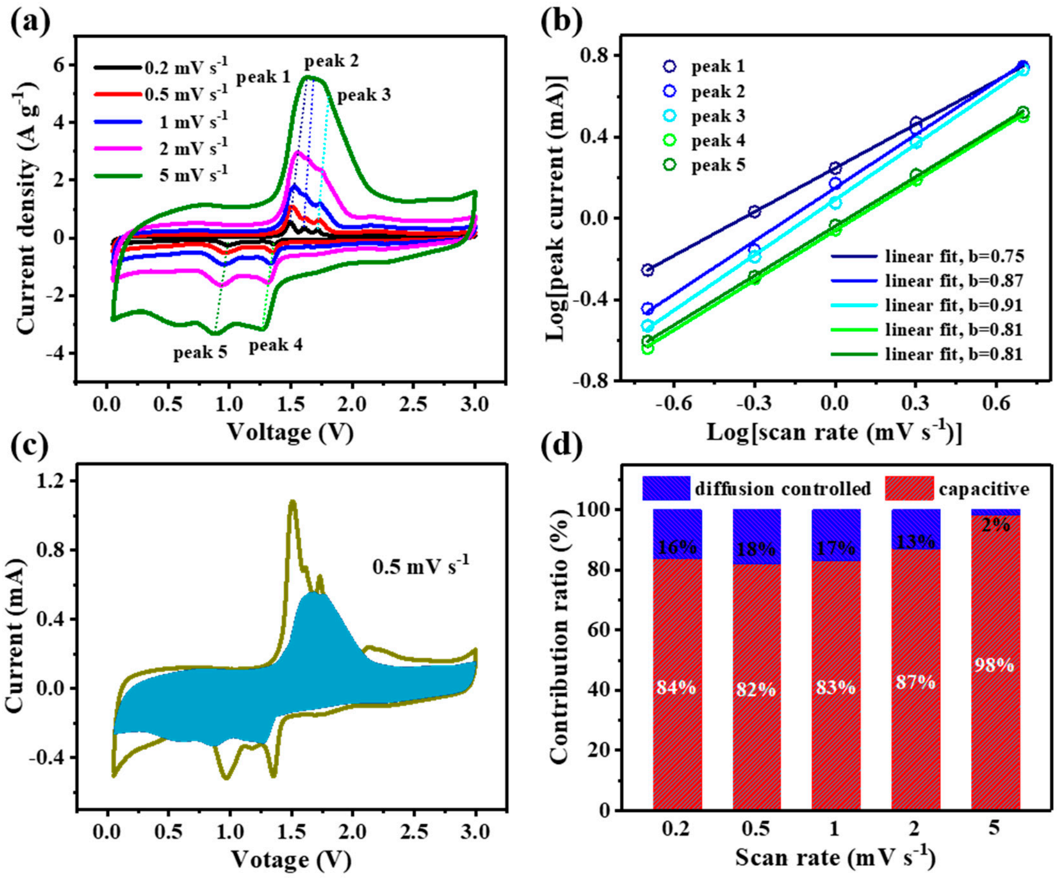

Figure S14. Kinetic analysis of Te NRs/rGO in SIBs half-cell: (a) CV curves at scan rates from 0.2 to 5.0 mV s^{-1}; (b) relationship between log *i* and log *v* plots; (c) the capacitive contribution at 0.5 mV s^{-1}; (d) capacitance contribution at various scan rates.

Table S1. Summary of nanorod length, width, and aspect ratio of Te NRs/rGO-310, Te NRs/rGO-620 and Te NRs/rGO-1240 composites

**Te NRs/rGO**	**nanorod length (nm)**	**nanorod width (nm)**	**Aspect ratio**
Te NRs/rGO-310	104.2 ± 12.2	10.4 ± 2.1	10.3 ± 1.6
Te NRs/rGO-620	166.5 ± 15.7	14.7 ± 2.5	11.7 ± 2.5
Te NRs/rGO-1240	169.4 ± 12.1	16.0 ± 2.2	10.7 ± 1.5

Table S2 Summary of the representative Nano-Te based electrode materials for SIBs

**Materials**	**Preparation**	**High rate capacity**	**Capacity retention**	**Ref.**
**Te NRs/rGO**	**50ºC, 3 h**	**338 mAh g^{-1} at 5 A g^{-1}**	**93.4% after 500 cycles at 5 A g^{-1}**	**This work**
Te/Cp	480ºC for 12 h	302 mAh g^{-1} at 0.8 A g^{-1}	68% after 1000 cycles at 3.2 A g^{-1}	[37]
Nano-Te@C	800ºC, 3 h	303 mAh g^{-1} at 0.8 A g^{-1}	90% after 1000 cycles at 0.06 A g^{-1}	[29]
NPCS/Te	480ºC for 6 h	382 mAh g^{-1} at 0.2 A g^{-1}	79.3% after 100 cycles at 0.2 A g^{-1}	[38]
Te@CMK-3	550ºC, 6 h	230 mAh g^{-1} at 0.8 A g^{-1}	98% after 95 cycles at 0.8 A g^{-1}	[36]
Te/CeO_2-QDs/HPC	480ºC for 20 h	260 mAh g^{-1} at 4 A g^{-1}	93.8% after 1000 cycles at 4 A g^{-1}	[34]
